# Parental communication on sexual and reproductive health issues and its associated factors among preparatory school students in Debre Tabor, Northcentral Ethiopia: institution based cross-sectional study

**DOI:** 10.1186/s13104-019-4644-y

**Published:** 2019-09-18

**Authors:** Maru Mekie, Wubet Taklual, Abenezer Melkie, Dagne Addisu

**Affiliations:** 1 Department of Midwifery, College of Health Science, Debre Tabor University, Debre Tabor, Ethiopia; 2 Department of Population Health, College of Health Science, Debre Tabor University, Debre Tabor, Ethiopia

**Keywords:** Communication, Sexual and reproductive health, School students, Debre Tabor, Ethiopia

## Abstract

**Objective:**

This study was aimed to assess parental communication on sexual and reproductive health (SRH) issues and its associated factors among preparatory school students in Debre Tabor, Northcentral Ethiopia. Institution based cross sectional study was employed among 394 preparatory school students through self-administered questionnaire.

**Result:**

A total of 394 students were participated in the study with a response rate of 100%. The magnitude of parental communication on SRH issues was found to be 68.5%, (95% CI (63.7, 72.8)). Low grade (AOR = 0.31, 95% CI (0.17, 0.58)), feel embraced to discuss about SRH issues (AOR = 0.31, 95% CI (0.17, 0.56)), and living with mother/father (AOR = 0.15, 95% CI (0.06, 0.36)) were associated with reduced parental communication on SRH. While, family size < 5 (AOR = 2.46, 95% CI (1.25, 4.84)), and believe on the importance of discussion on SRH (AOR = 10.83, 95% CI (5.07, 23.17) were found to be associated with increased communication about SRH issues. Health education shall be given to preparatory school students on importance of discussion on SRH issues and related consequences of risky sexual behaviors.

## Introduction

World Health Organization (WHO) defines adolescent and youth population are persons whose age is found between 10–19 years and 15–24 years respectively [1]. Youth population has reached 1.8 billion in the world in which the majority resides in developing world. Despite being the healthiest group of the general population, 1.3 million youth population (aged 15–24 years) reported to die annually from preventable causes of death [2].

Adolescence is a critical period of human development often characterized by confusion, mixed interpretation and understanding of adult behavior, especially with drugs, alcohol, and sex which are the most common problems associated with sexual maturation [3].

Adolescent health is the most ignored area in health priority. Shortage of youth-friendly services and lack of integration are common problems in most developing countries [4]. Young people usually engage in risky sexual behaviors which have adverse health outcome including unintended pregnancy and sexually transmitted diseases [5]. Effective education on sexuality for young people is lacking in many countries. A global measure of coverage related to sexual education indicated that only 36% and 24% of youth male and female respectively in developing regions have comprehensive and correct knowledge of Human Immune Deficiency Virus (HIV)/Acquired Immune Deficiency Syndrome (AIDS) [6, 7].

Poor SRH accounts for a third of the global burden of illness and early death among women of reproductive age. Adolescents’ SRH is strongly linked with social, cultural, and economic environment [4, 8]. Young people are a population group highly affected by the burden of unwanted pregnancy, HIV/AIDS, sexual transmitted infections (STIs) and other reproductive ill health due to lack of awareness about risky sexual behaviors [6, 9]. Pregnancy and childbirth complications are the leading cause of death among girls aged 15–19 years globally, the majority being in low income countries [10].

Parent–children communication with regards to sexuality is critical in informing youth about risk and protective behaviors, and decrease the likelihood of involvement in risky sexual behaviors [11, 12]. However, parental communication regarding SRH issues is lacking despite parents are the primary sources of information regarding sexuality [13–15]. Different factors can affect parental communication on SRH issues. Studies indicated that fear of parent, cultural taboos attached to sex, embarrassments, parents’ level of education, poor knowledge of parents about SRH, age, and grade level of the participants were found to be the determinants of parent–children communication on SRH [15, 16].

Despite the government of Ethiopia established adolescent and youth health strategy, reproductive health problems are continue to be a challenge among young people [17]. Parental communication on SRH issues is imperative in reducing risky sexual behaviors [7, 18, 19]. Nonetheless, evidence is lacking regarding parental communication and its associated factors in the study area. Hence, this study was aimed to assess parental communication on SRH issues among preparatory students in Debre Tabor.

## Main text

### Methods

#### Study setting and design

The study was conducted in Tewodros II preparatory school, Debre Tabor, Northcentral Ethiopia which is located about 667 km from the capital city Addis Ababa. There were 2094 students in Tewodros II preparatory school who follow their education in 2019 of whom 1092 were grade 11 and 1002 were grade 12. Institution based cross sectional study was applied from March 28–April 4, 2019.

#### Study population

All Tewodros II preparatory school students who follow their education during the study period were the study population. Preparatory school students who attend their education in the regular program were included. While, students who absent during the time of data collection period were excluded in the study.

#### Sample size and sampling procedures

The sample size was determined by using a single population proportion formula with margin of error (w) 5%, 95% confidence level, and adding 10% non-response. The proportion of communication on SRH issues was taken to be 36.9% from a previous study in Debre Markos [20]. The final sample size after adding 10% non-response rate was 394.

Initially stratification was made by grade (11th and 12th). Then to select the study subjects from each section, proportionate to size technique was used. Finally, simple random sampling technique was used to select study participants in each section by using students’ roster as a sampling frame.

#### Data collection procedures and quality assurance

A structured self-administered questionnaire was used to collect the data. The questionnaire was prepared in English and translated to Amharic language for easiness and translated back to English to check consistency. Translation of questionnaire was done by language experts in both cases. The questionnaire was adapted and modified from review of different literatures [9, 16, 21].

The quality of data was maintained by using structured and pretested questionnaire. Two days training was given for facilitators and supervisors about the data collection processes. Necessary amendments were made on the study tools after doing pertest on 5% of the sample. Strict supervision and monitoring were also performed during the data collection period by supervisors and investigators.

#### Measurements

Those who discussed at least two issues on SRH (condom, STIs, HIV/AIDS, sexual intercourse, menstruation, unwanted pregnancy, contraception) with their parents in the last 12 months were considered as communicated on SRH issues [9]. Parent was defined as a biological parent or a guardian who lived with and took care of the adolescent/youth [22].

#### Data processing and analysis procedures

The data were entered in a template prepared in EpiData version 3.1. Then the data were exported to SPSS version 20 for cleaning and analysis. Descriptive statistics such as frequency and other statistical summary measures were used to describe the study participants. A bivariable and multivariable logistic regression analysis were used to identify factors associated with outcome variable. Variables with P value of < 0.2 were entered in the multivariable logistic regression analysis. An adjusted odds ratio at 95% confidence level were used to identify the significance association. A P-value of < 0.05 was used to determine the significance of association.

#### Ethical consideration

Ethical clearance and letter of permission were obtained from Debre Tabor University Collage of Health Sciences. Then the permission letter was submitted to Tewodros II preparatory school officials to contact the students. Written consents was obtained from the study participants after explaining the study objectives and procedures. For this purpose, a one-page consent letter was attached to the cover-page of each questionnaire. Privacy and confidentiality were maintained throughout data collection period.

### Results

#### Sociodemographic characteristics of the study participants

A total of 394 of respondents completed the questionnaire giving a response rate of 100%. With regards to sex distribution, more than half, 210 (53.3%) were males and 184 (46.7%) were females. The majority, 290 (73.6%) of the students were within the age group of 15–19 years. The mean age of respondents was 19.33 ± 1.72 years with a minimum and maximum age of 17 and 24 years respectively. The majority of the study participants, 386 (98.0%) were orthodox Christian followers. While, 8 (2.0%) of the study participants were Muslim religion followers. The mean household income of family of the study participants’ was 3800.81 ± 2661.03 Ethiopian birr (Table 1).

**Table 1:** Socio-demographic characteristics of Tewodros II preparatory school students, Northcentral Ethiopia, 2019

Variables	Frequency (n)	Percent (%)
Age		
15–19 years	290	73.6
20–24 years	103	26.4
Grade		
11th	182	46.2
12th	212	53.8
Stream		
Natural	247	62.7
Social	147	37.3
Sex		
Male	210	53.3
Female	184	46.7
Religion		
Orthodox	386	98.0
Muslim	8	2.0
With whom student live		
With parents	371	94.2
Live alone	23	5.8
Education status of mother		
Illiterate	107	27.2
Read and write	122	31.0
Primary	77	19.5
Secondary and above	88	22.3
Family size of your parent		
1–4	140	35.5
5–9	241	61.2
≥ 10	13	3.3
Occupation of the mother		
Housewife	283	71.8
Government employee	30	7.6
Private employee	15	3.8
Merchant	66	16.8
Household monthly income		
< 3333	195	49.5
≥ 3333 ETB	199	50.5

#### Communication on SRH issues

The magnitude of parental communication on SRH issues was found to be 68.5%, (95% CI (63.7, 72.8)). With regards to significance of discussing about SRH issues, the majority, 305 (77.4%) reported that it is important to discuss about SRH issues with parents. On the other hand, 56 (14.2%) of the study participants did not accept the importance of discussing SRH issues with parents. With regards to preference to discuss about SRH issues, 164 (41.6%), 103 (26.1%), and 51 (12.9%) of the study participants prefer to discuss SRH issues with friends, mother, and father respectively (Table 2).

**Table 2:** Communication on SRH issues among Tewodros II preparatory school students, Northcentral Ethiopia, 2019

Variable	Frequency (n)	Percent (%)
Discuss on SRH issues		
Yes	270	68.5
No	124	31.5
Discussion on SRH is important		
Yes	305	77.4
No	56	14.2
Don’t know	33	8.4
Preference to discuss about SRH		
Father	51	12.9
Mother	103	26.1
Brother/sister	34	8.6
Girl/boy friends	164	41.6
Teacher	42	10.7

#### Factors associated with communication on SRH issues

Participants’ grade (AOR = 0.31, 95% CI (0.17, 0.58)), feel embraced to discuss about SRH (AOR = 0.31, 95% CI (0.17, 0.56)), family size < 5 (AOR = 2.46, 95% CI (1.25, 4.84)), believe on the importance of discussion on SRH (AOR = 10.83, 95% CI (5.07, 23.17), family condition they live with (AOR = 0.15, 95% CI (0.06, 0.36)) were found to be significantly associated with communication on SRH issues with parents (Table 3).

**Table 3:** Multivariable analysis of factors associated with communication on SRH issues among preparatory school students in Debre Tabor, Northcentral Ethiopia, 2019

Variables	Discuss on SRH issues		COR (95% CI)	AOR (95% CI)
	NO N (%)	Yes N (%)		
Religion of the study participants				
Orthodox	114 (95.0)	272 (99.3)	6.81 (1.36, 34.25)^a^	0.82 (0.13, 5.18)
Muslim	6 (5.0)	2 (0.7)	1	1
Stream of the study participants				
Natural	66 (55.0)	181 (66.1)	1.54 (1.00, 2.38)	0.64 (0.34, 1.19)
Social	54 (45.0)	93 (33.9)	1	1
Grade of the study participants				
11th	67 (55.8)	115 (42.0)	0.60 (0.39, 0.92)^a^	0.31 (0.17, 0.58)^b^
12th	53 (44.2)	159 (58.0)	1	1
Feel embraced to discuss SRH issues				
Yes	80 (66.7)	124 (45.3)	0.38 (0.24, 0.60)^a^	0.31 (0.17, 0.56)^b^
No	40 (33.3)	150 (54.7)	1	1
Family size				
1–4	24 (20.0)	116 (42.3)	2.59 (1.58, 4.22)^a^	2.46 (1.25, 4.84)^b^
5 and more	96 (80.0)	158 (57.7)	1	1
Do you think discussing about SRH issue is important				
Yes	71 (57.3)	234 (86.7)	1.97 (1.23, 3.16)^a^	10.83 (5.07, 23.17)^b^
No	53 (42)	36 (13.3)	1	1
Occupation of the mother				
Housewife	95 (76.6)	191 (69.7)	1.06 (0.60, 1.86)	1.13 (0.45, 2.84)
Government employee	5 (4.0)	25 (9.1)	2.67 (0.90, 7.92)	3.71 (0.97, 14.12
Private employee	1 (0.8)	15 (5.5)	7.49 (0.93, 60.60)	11.53 (1.20, 110.45)^b^
Merchant	23 (18.5)	43 (15.7)	1	1
Currently live with				
Mother and father	104 (86.7)	177 (66.5)	0.18 (0.08, 0.40)^a^	0.15 (0.06, 0.36)^b^
Mother only	8 (6.7)	15 (5.6)	0.16 (0.05, 0.51)^a^	0.03 (0.01, 0.15)^b^
Father only	1 (0.8)	7 (2.6)	0.73 (0.08, 6.84)	0.72 (0.07, 7.60)
Live alone	7 (5.8)	67 (25.2)	1	1

1: reference

^a^Significant in the bivariable analysis

^b^Significant in the multivariable analysis

### Discussion

This study was aimed to assess communication on SRH issues among Tewodros II preparatory school students. The magnitude of parental communication on SRH issues was reported to be (68.5%, 95% CI (63.7, 72.8)). Parent–child communication promotes adolescents’ self-esteem and prevents risky behaviors [7]. The finding of this study is found to be lower than a study conducted among preparatory school students in Hayik, Northeast Ethiopia in which 83% of the study participants reported to discussed SRH issues [16]. The difference might be related to difference in level of awareness and culture related to openness related to SRH issues.

The odds of having communication on SRH issue were found to be lower among grade 11 students (AOR = 0.31, 95% CI (0.17, 0.58)) compared to their grade 12 counterparts. This might be related to difference in maturity and level of awareness as the grade level increases. The finding of this study is supported by a previous study conducted in Northeast Ethiopia [16].

Feeling embraced to communicate SRH issues was found to be significantly associated with parental communication on SRH issues. Study participants who feel embraced were less likely to discuss SRH issues with partners compared with counterparts (AOR = 0.31, 95% CI (0.17, 0.56)). This finding indicated that parents are not open to their children in discussion related to SRH which leads young people to feel embraced in discussion of sexual related issues. The finding of this study is consistent with studies conducted in Hawassa, Ethiopia [23] and Johannesburg, South Africa [21]. In the same manner, family size where the study participants live was found to be significantly associated with discussion on SRH issues. The odds of having communication on SRH issue were found to be 2.46 times higher among participants live in family of < 5 compared with counterparts (AOR = 2.46, 95% CI (1.25, 4.84)). The finding of this study indicated that those parents with small family size had better chance of discussing SRH issues with their children. Poor parental communication is barrier for discussion related to SRH issues [24].

Participants who had positive belief about the importance of communication on SRH issues were 10.83 times more likely to discuss on SRH issues compared to counterparts (AOR = 10.83, 95% CI (5.07, 23.17)). The finding of this study is similar with a previous study conducted in Woldia, Northeastern Ethiopia [9]. The finding of our study implied that young people are not open enough to discuss about SRH issues due to poor understanding of the significance of discussion. Likewise, family condition was found to be statistically significant with discussion on SRH issues. The odds of discussing on SRH issues were found to be lower among participants who live with mother/father compared with those who live alone (AOR = 0.15, 95% CI (0.06, 0.36)). This finding indicated that those who live alone had better decision making power, being open and have confidence with regards to SRH issues [22]. Skills related to social and emotional competence promote positive social development which assists youth in developing close friendships and having positive peer relations [25].

### Conclusion and recommendation

The magnitude of discussion on SRH issues was found to be low. Participants’ grade, feel embraced to discuss SRH, family size, believe on importance of discussion on SRH issues, and family condition of the participants were found to be significantly associated with discussion on SRH. Health education shall be given to preparatory school students on importance of discussion on SRH issues and related consequences of risky sexual behaviors.

## Limitations of the study

The study was conducted at facility level which could not be generalized for the general youth population. Cross-sectional study design was used which might affect cause effect relationship. Structured and pretested tool was used for data collection which could be taken as a strength.

## Data Availability

The datasets used in this study are available from the corresponding author and can be accessible through reasonable request.

## Abbreviations

AIDS: acquired immune deficiency syndrome
AOR: adjusted odds ratio
COR: crude odds ratio
CI: confidence interval
ETB: Ethiopian birr
HIV: human immune deficiency virus
SRH: sexual and reproductive health
STIs: sexual transmitted infections
